# Primary High-Grade Diffuse Large B-Cell Lymphoma of the Cecum: A Report of a Rare Case

**DOI:** 10.7759/cureus.99277

**Published:** 2025-12-15

**Authors:** Azhar K Adhad, Zuhair D Hammood, Omar Sultan, Waleed N Hosi, Sarhang S Abdalla, Harun A Ahmed, Berun Abdalla

**Affiliations:** 1 Gastroenterology and Hepatology, Nursing Home Hospital, Medical City Complex Bab Al Muadham, Baghdad, IRQ; 2 Surgical Oncology, Tikrit Teaching Hospital, Tikrit, IRQ; 3 Scientific Research, Kscien Organization for Scientific Research (Middle East Office), Sulaymaniyah, IRQ; 4 Radiology, Tikrit University, Tikrit, IRQ; 5 Pathology, Tikrit University, Tikrit, IRQ; 6 Scientific Affairs, Smart Health Tower, Sulaymaniyah, IRQ

**Keywords:** cecal mass, cecum, colorectal tumors, diffuse large b-cell lymphoma, non-hodgkin lymphoma

## Abstract

Primary diffuse large B-cell lymphoma (DLBCL) of the colon is rare and most commonly involves the cecum due to its rich lymphoid tissue. Diagnosis is often delayed because symptoms are nonspecific and endoscopic biopsies may be inconclusive. We report a case of high-grade cecal DLBCL in a 22-year-old male patient who presented with right-sided abdominal pain, weight loss, intermittent fever, and night sweats over three months. Examination revealed a palpable right iliac fossa mass, and imaging demonstrated a large cecal lesion with regional lymphadenopathy; however, colonoscopic biopsies were nondiagnostic. The patient underwent right hemicolectomy, and histopathology with immunohistochemistry confirmed high-grade DLBCL (CD20+, CD10+, BCL6+, Ki-67 ≈ 80%). Postoperatively, he developed a wound infection that was managed conservatively before initiating chemotherapy. This case illustrates that, despite its rarity and diagnostic challenges, cecal DLBCL can be successfully treated through a multidisciplinary approach. Clinicians should consider lymphoma in the differential diagnosis of cecal masses in young adults, particularly when B symptoms are present or biopsy results are inconclusive.

## Introduction

Diffuse large B-cell lymphoma (DLBCL) may arise in nodal or extranodal sites, with the gastrointestinal tract representing the most frequent extranodal location. Approximately 10-15% of all non-Hodgkin lymphoma (NHL) and 1-4% of gastrointestinal neoplasms originate within the digestive system [1]. Despite this, primary colorectal involvement is distinctly uncommon, accounting for only 0.1-0.6% of colorectal malignancies and around 3% of gastrointestinal lymphomas [2].

According to the fifth edition of the World Health Organization (WHO) Classification of Tumors of Hematopoietic and Lymphoid Tissues, lymphoid malignancies encompass more than 50 well-defined subtypes [3]. These entities range from indolent neoplasms, such as follicular lymphoma, to highly aggressive forms. Within this spectrum, DLBCL stands out as the most common aggressive subtype of NHL, reflecting its wide biological diversity and substantial clinical impact [4].

Among colorectal lymphoid tumors, DLBCL constitutes the predominant histological subtype. Reported cases show a slight male predominance, with peak incidence in the sixth and seventh decades of life, although presentations across a wider age range have also been documented [5]. The cecum is frequently cited as the most commonly affected region, likely due to its abundant lymphoid tissue [6]. Nevertheless, primary lesions have been described throughout the colon, including the ascending, descending, sigmoid, and rectosigmoid segments, and rare involvement of anorectal structures such as hemorrhoidal tissue has also been reported [7].

Epidemiologic analyses highlight the heterogeneous nature of colorectal DLBCL, with variations in demographic patterns, anatomical distribution, and biological characteristics. Recent studies underscore the prognostic relevance of factors such as lactate dehydrogenase levels, tumor location, and disease stage in predicting outcomes [4].

The aim of this report is to present and characterize a rare diagnosis of high-grade DLBCL of the cecum. All referenced sources were critically assessed for reliability [8].

## Case presentation

A 22-year-old male patient with no significant past medical or family history presented with a three-month history of abdominal pain predominantly localized to the right hypochondrium. During this period, he reported nausea, intermittent vomiting, reduced appetite, intermittent fever with drenching night sweats, and an unintentional weight loss of approximately 8 kg over two months.

On examination, the patient was alert, oriented, and hemodynamically stable, though he appeared pale and fatigued. Abdominal evaluation revealed a prominent palpable mass with mild tenderness in the right iliac fossa, along with mild tenderness in the left hypochondrium. No other significant abnormalities were noted. Vital signs were within normal limits, including a temperature of 36°C and blood pressure of 110/70 mmHg; his body weight was 60 kg.

Laboratory evaluation demonstrated mild leukocytosis with neutrophilia and thrombocytosis. Inflammatory markers were elevated, with an erythrocyte sedimentation rate (ESR) of 65 mm per hour and a C-reactive protein (CRP) level of 44 mg per liter. Liver, renal, and thyroid function tests were normal, as were HbA1c (5.3%) and anti-tissue transglutaminase (tTG) IgA and IgG serology (Table 1). Electrocardiogram (ECG) and echocardiography showed no abnormalities.

**Table 1:** Laboratory test results ESR: erythrocyte sedimentation rate; CRP: C-reactive protien; AST: aspartate aminotransferase; ALT: alanine transaminase; ALP: alkaline phosphatase; TSH: thyroid stimulating hormone; tTG: tissue transglutaminase

Tests	Patient Values	Reference Ranges
White Blood Cell Count	13.2 ×10⁹/L	4.0–11.0 ×10⁹/L
Neutrophils	9.8 ×10⁹/L (74%)	2.0–7.0 ×10⁹/L (40–70%)
Platelets	520 ×10⁹/L	150–450 ×10⁹/L
ESR	65 mm/hr	< 20 mm/hr
CRP	44 mg/L	< 5 mg/L
AST	28 U/L	10–40 U/L
ALT	32 U/L	7–56 U/L
ALP	110 U/L	44–147 U/L
Blood Urea	28 mg/dL	15–48 mg/dL
Serum Creatinine	0.9 mg/dL	0.7–1.3 mg/dL
TSH	2.1 µIU/ml	0.8-6.0 µIU/ml
HbA1c	5.3%	4.0–5.6%
Anti-tTG IgA	3 U/mL	< 4 U/mL
Anti-tTG IgG	2 U/mL	< 6 U/mL

**Figure 1:**
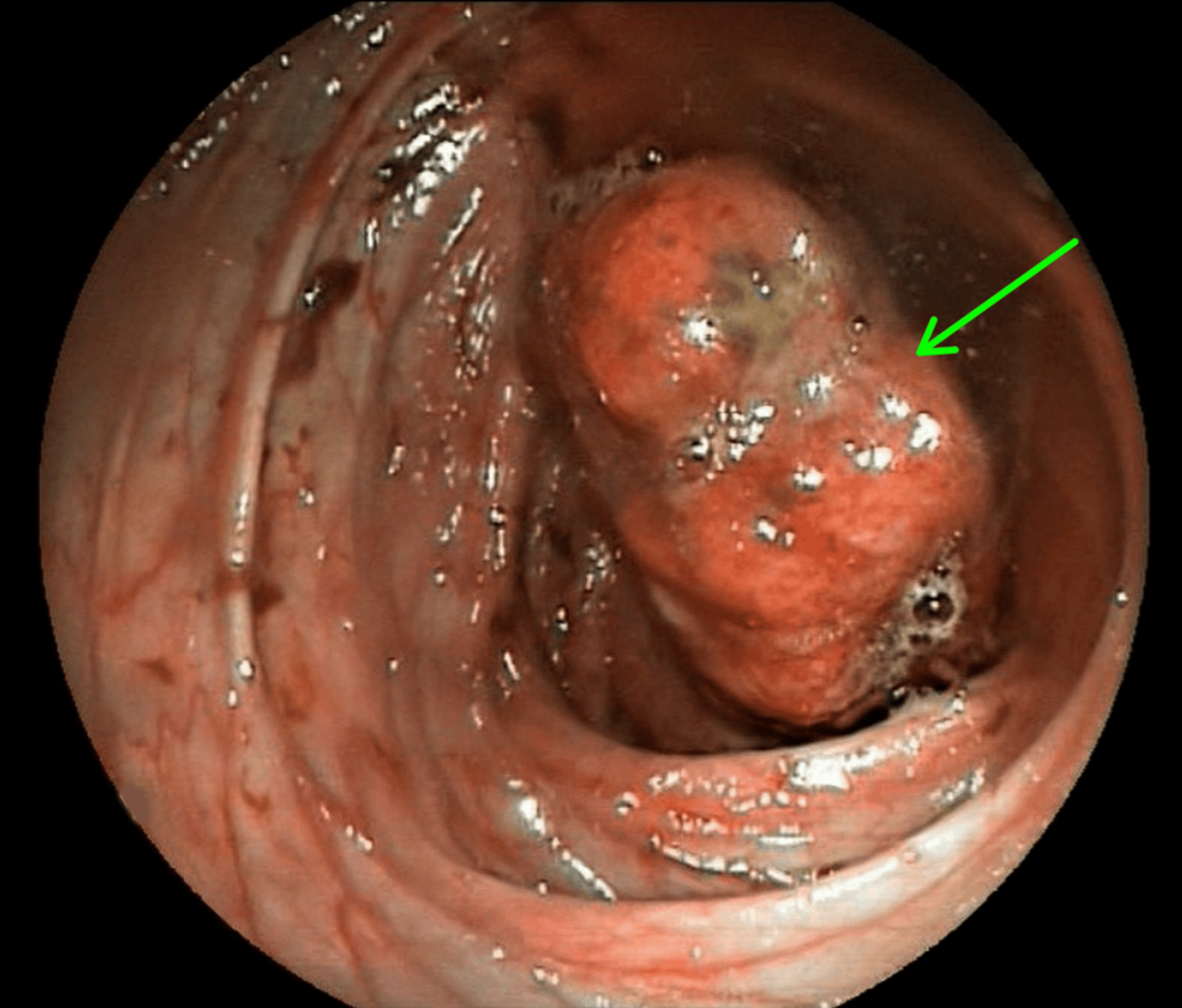
Endoscopic view demonstrating a large, elongated, fleshy polypoid mass arising from the ileocecal valve and occupying the cecal lumen, with upward extension into the distal ascending colon. The lesion, measuring approximately 4 × 3 cm, shows central mucosal ulceration and exhibits a characteristic to-and-fro motion within the lumen. The green arrow indicates the ulcerated focal area on the surface of the mass.

**Figure 2:**
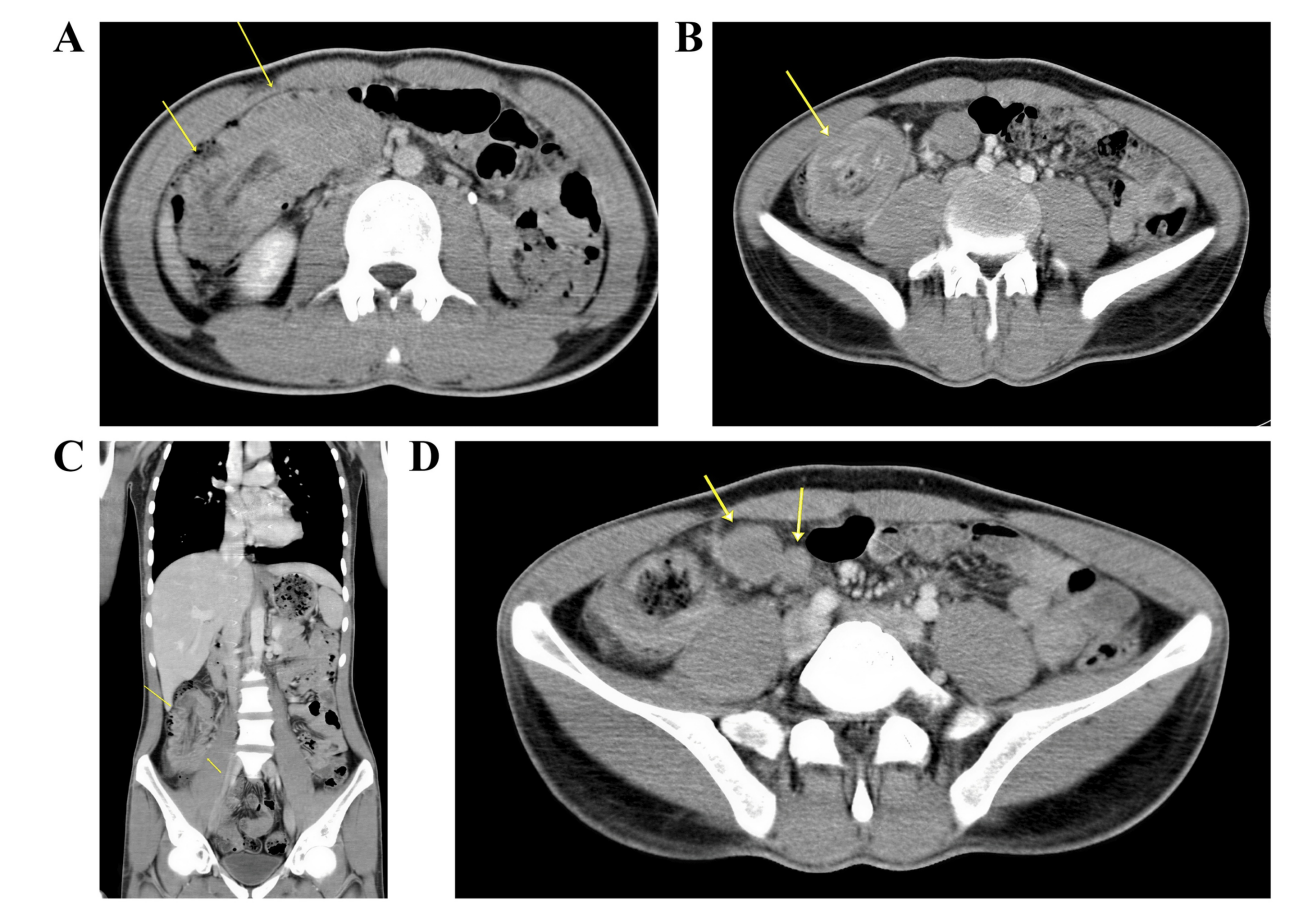
Contrast-enhanced CT scan (A) Axial image showing aneurysmal dilatation of the cecum with diffuse, marked mural thickening (yellow arrows). (B) Axial view demonstrating a mass-like concentric mural thickening involving the ileocecal region (yellow arrow). (C) Coronal reconstruction showing segmental mural thickening of the terminal ileum and cecum (yellow arrow). (D) Axial image displaying enlarged locoregional mesenteric lymph nodes (yellow arrows).

Abdominal ultrasonography revealed mucosal thickening involving the terminal 20 cm of the ileum and the cecum. Colonoscopy demonstrated normal mucosa and vascularity; however, a large, elongated intraluminal mass extending from the cecum to the distal ascending colon near the hepatic flexure was identified, causing partial luminal obstruction (Figure 1). Complete visualization of the cecum was not possible due to the obstructing mass, and biopsies obtained were inconclusive. Computed tomography (CT) imaging confirmed a localized mass with regional lymphadenopathy (Figure 2).

Due to the obstructive symptoms and radiologic suspicion of malignancy, the case was discussed in a multidisciplinary team meeting, which recommended surgical resection. The patient subsequently underwent a right hemicolectomy with omentectomy under general anesthesia.

**Figure 3:**
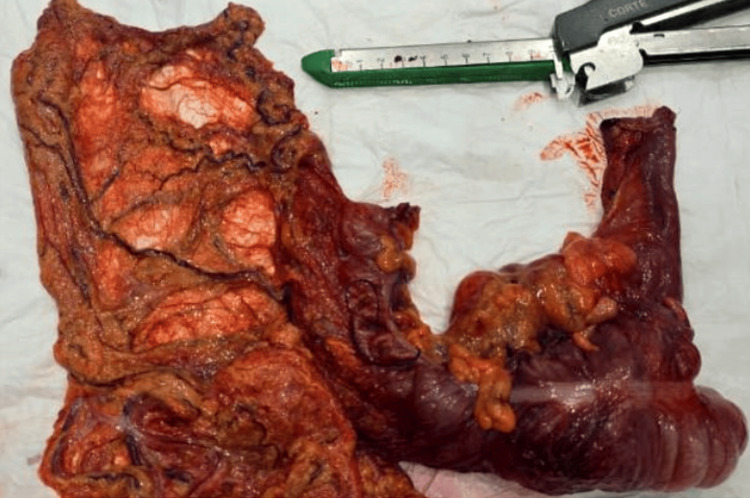
Right hemicolectomy specimen showing a bulky polypoid cecal mass extending into the ascending colon. The mass measures approximately 5 × 4 × 4 cm and demonstrates deep mural thickening with adjacent mesenteric fat involvement.

Gross examination of the resected specimen, which included the cecum, part of the ascending colon, and the terminal ileum, revealed a polypoid cecal mass measuring 5 by 4 by 4 cm within an 8 by 5 cm segment, with deep mural thickening and areas of necrosis (Figure 3). The proximal and distal margins were free of tumor, and three enlarged pericolic lymph nodes up to 2 cm were identified.

Microscopically, the mass showed diffuse infiltration by large atypical lymphoid cells replacing the mucosa, submucosa, and muscularis propria. The neoplastic cells exhibited vesicular nuclei, prominent nucleoli, frequent mitoses, and extensive necrosis with overlying mucosal ulceration. The regional lymph nodes were completely replaced by similar malignant cells, confirming nodal involvement, while the omentum was free of disease.

Final diagnosis was high-grade NHL (DLBC Type) involving the cecum with regional lymph node involvement, stage IIA, localized extranodal lymphoma (gastrointestinal tract being the primary site. 

**Figure 4:**
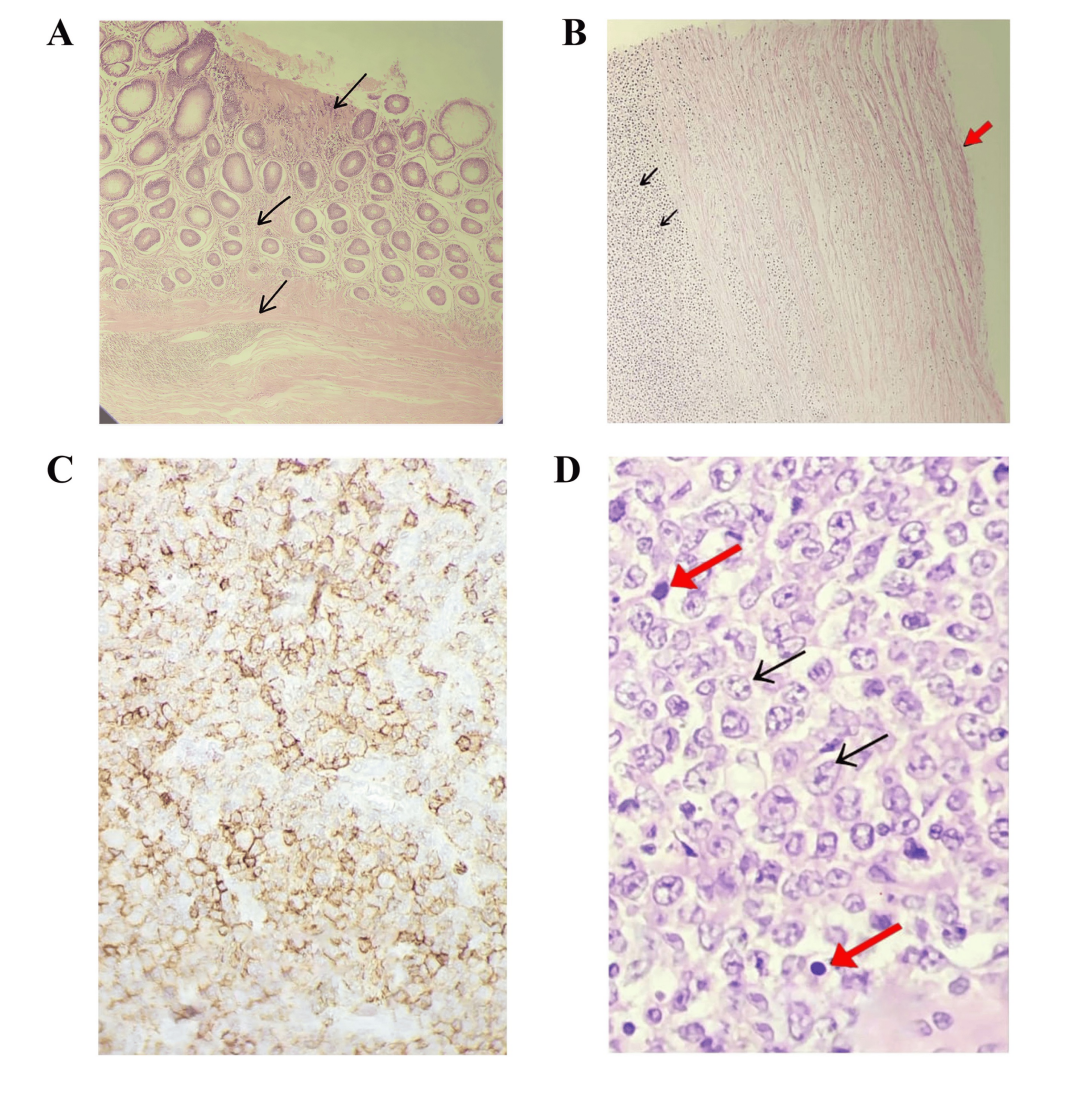
Histology findings (A) Section from a cecal mass showing an ulcerated area with diffuse infiltration of the mucosa and submucosa by malignant lymphoid cells (black arrow) (H&E, magnification x 40); (B) Ulcerated area showing complete loss of mucosa (red arrow) and diffuse infiltration of the submucosa by malignant lymphoid cells (double black arrow) (H&E, magnification x 40); (C) Section from a cecal mass and the pericecal lymph node showing large malignant cells with diffuse infiltration by atypical lymphoid cells exhibiting vesicular chromatin, prominent nucleoli (black arrow), and abundant mitotic figure (red arrow) (H&E, magnification x 100). (D) Section of immunohistochemistry stain (CD20) showing a strong, homogeneous membranous staining pattern in the vast majority of malignant lymphoid cells.

Immunohistochemistry demonstrated strong positivity for CD20, CD10, and BCL6, with negativity for CD3, consistent with B-cell lineage (Figure 4). The Ki-67 proliferation index was approximately 80%, indicating high proliferative activity. These findings confirmed the diagnosis of high-grade DLBCL of the cecum with regional lymph node involvement, corresponding to Stage IIA localized extranodal lymphoma.

Postoperatively, the patient developed a wound infection associated with a low-output fistula, which was managed conservatively. He was then referred to the clinical haematology service and commenced an appropriate chemotherapy protocol, with planned follow-up to monitor treatment response. 

A chronological summary of the patient’s clinical course, investigations, and progression is presented in Table 2.

**Table 2:** Timeline of the patient’s clinical course CBC: complete blood count; CRP: C-reactive protien; MDT: multi-disciplinary team

Time Period	Event/Clinical Course
3 months before admission	Onset of abdominal pain in the right hypochondrium
2–3 months before admission	Development of nausea, intermittent vomiting, reduced appetite
2 months before admission	Unintentional weight loss of 8 kg, intermittent fever, drenching night sweats
At presentation	Pale, fatigued appearance; palpable mass in the right iliac fossa
Day 0 – Initial investigations	CBC abnormalities; ESR 65 mm/hour; CRP 44 mg/L; normal organ function tests
Day 3 – Ultrasonography	Mucosal thickening of terminal ileum and cecum
Day 7 – Colonoscopy	Large intraluminal mass extending from cecum to distal ascending colon; biopsies inconclusive
Day 10 – CT scan	Localized mass with regional lymphadenopathy
Day 14 – MDT meeting	Surgical resection recommended
Day 20 – Surgery	Right hemicolectomy with omentectomy performed
Postoperative period	Wound infection and low output fistula; managed conservatively
4–6 weeks postoperative	Referral to clinical haematology
Follow-up	Initiation of chemotherapy protocol and ongoing monitoring

## Discussion

Primary colorectal DLBCL is exceedingly rare, accounting for less than 1% of colorectal malignancies and approximately 0.3-0.6% in some series [9]. Although the gastrointestinal tract is a common extranodal site for NHL, colorectal involvement is especially uncommon. There is a slight male predominance, as most colorectal DLBCL cases occur in the sixth to seventh decades of life [5,6]. This makes presentation at 22 years of age particularly noteworthy, falling far outside the expected epidemiologic range. Known predisposing factors include HIV/AIDS, inflammatory bowel disease, celiac disease, and other causes of immunosuppression [8]. In the present case, the patient was a 22-year-old male without risk factors, further highlighting the unusual nature of this diagnosis. Notably, the tumor originated in the cecum, the most frequently involved segment due to its lymphoid tissue abundance.

Patients typically present with nonspecific gastrointestinal symptoms such as abdominal pain, unintentional weight loss, and altered bowel habits, including intermittent diarrhea or constipation. These vague symptoms often delay diagnosis, as lymphoma is not initially suspected [6]. Our patient's abdominal pain and weight loss align with these typical symptoms, yet what distinguishes this case is the presence of systemic “B” symptoms, including recurrent fever and night sweats, features rarely observed in primary colorectal lymphoma, with one study reporting none among 18 patients [5]. Physical examination commonly reveals a palpable abdominal mass [6], consistent with the firm mass identified clinically. Despite the tumor’s large size, acute obstruction was absent, consistent with the known absence of desmoplastic reaction in lymphomas [9], and explaining only partial luminal narrowing.

Contrast-enhanced CT remains the most informative modality, usually showing a bulky mass with lymphadenopathy. This was observed radiologically in our patient. However, imaging overlaps with adenocarcinoma and inflammatory disease [10], and rare presentations such as intussusception or appendicitis-like features have been reported [5]. Colonoscopy is a standard diagnostic tool; however, mucosal biopsies may be nondiagnostic when infiltrative growth is predominantly submucosal. This limitation occurred here, where endoscopic biopsies failed to detect lymphoma, making surgical resection a diagnostic necessity, a pattern echoed in multiple prior reports.

Definitive diagnosis relies on histopathology and immunophenotyping. Our patient demonstrated diffuse infiltration by large atypical cells, with a high Ki-67 index (~80%) and immunophenotype CD20+, CD10+, BCL6+, confirming high-grade DLBCL [11]. A striking feature in this case is MYC-positive expression, reflecting high-risk biology and rapid proliferation, which contrasts with many previously documented colorectal cases that lacked MYC expression and presented with less aggressive behavior [5,7]. Primary colorectal lymphomas are predominantly DLBCL, with other subtypes distinctly uncommon [6].

A lymphoma is classified as primary to the gastrointestinal tract when the bowel lesion is dominant despite nodal involvement [6]. Our patient fit this definition, presenting as Stage IIA with regional node involvement. In comparison to reported cases, which usually involve older patients with slower progression and without B symptoms, this case represents a uniquely aggressive phenotype at an unusually young age. This highlights an important learning point: DLBCL should not be excluded based solely on patient age.

Due to its rarity, management strategies mirror those of nodal DLBCL rather than standardized colorectal protocols [2]. Multidisciplinary consensus favors resection followed by R-CHOP (rituximab, cyclophosphamide, hydroxydaunorubicin (Doxorubicin), oncovin (Vincristine), and prednisone/prednisolone) chemotherapy [6], the same approach implemented in our case. Three comparable case reports describe favorable outcomes following similar intervention, yet most involved older patients without MYC-positive disease or systemic symptoms, reinforcing the value of this report in expanding clinical awareness [1,2,6].

## Conclusions

This case highlights that, despite rarity and initial diagnostic challenges, primary high-grade cecal DLBCL can be effectively managed with a multidisciplinary approach, supporting potential cure or long-term remission.
